# Cytokinin‐regulated targets of Cytokinin Response Factor 6 are involved in potassium transport

**DOI:** 10.1002/pld3.291

**Published:** 2020-12-08

**Authors:** Ariel M. Hughes, Paul J. Zwack, Paul A. Cobine, Aaron M. Rashotte

**Affiliations:** ^1^ Department of Biological Sciences Auburn University Auburn AL USA

**Keywords:** CRF6, Cytokinin, Cytokinin Response Factor, potassium, transport

## Abstract

Cytokinin (CK) is a plant hormone crucial to plant development and growth. Cytokinin Response Factor 6 (CRF6) is a CK‐induced transcription factor that is part of the CK signaling cascade. While the role of CRF6 has been examined in oxidative stress response, there has been surprisingly little investigation of CRF6 in the context of CK signaling, including identifying CK‐regulated targets of CRF6. Here, we conduct a transcriptomic study of Arabidopsis examining the CRF6 mutant (*crf6*) in the presence and absence of CK, revealing 163 downstream CRF6‐dependent CK‐regulated differentially expressed genes (DEGs). 15.3% of these DEGS were found as overlapping with larger number of standardly identified CK‐regulated DEGs, suggesting that CRF6 is involved in regulating a subset of downstream CK responses through these gene targets. The general transcriptional regulation of CRF6‐dependent CK‐regulated DEGs indicates that CRF6 may function as a negative regulator of CK response. We investigated one subset of CRF6 CK‐dependent targets (*SKOR*, *HAK5,* and *NRT1. 5*) involved in an underexamined functional role of CK response: the uptake and transportation of potassium. To determine how CK and CRF6 are involved in potassium acquisition and distribution, ionomic and physiological experiments were conducted on plants grown in media with sufficient and deficient potassium concentrations and in the presence and absence of CK. In order to investigate how CK alone affects potassium transport, similar experiments were performed on *skor, hak5,* and *nrt1.5* mutant lines of these CRF6‐dependent CK‐regulated targets. These findings indicate novel connections between CK and potassium transport, which appear to be regulated in a CRF6‐dependent manner.

## 1 INTRODUCTION

Cytokinin (CK) is an important plant hormone involved in growth, development, and response to environmental stimuli (Mok & Mok, 2001; Werner & Schmulling, 2009). It is involved in the regulation of leaf senescence, root cell division, shoot cell elongation, formation of lateral roots, protection from pathogens, and plays a role in response to nutrient deprivation (Argueso et al., 2009; Böttger, 1974; Kieber & Schaller, 2014; Zwack et al., 2013). Initial regulation of these varied roles is carried out through the two‐component CK signaling pathway (TCS). One branch of the TCS leads to a set of transcription factors called Cytokinin Response Factors (CRFs). CRFs were first identified from an examination of genes induced in *Arabidopsis thaliana* treated with exogenous CK (Rashotte et al., 2003; Rashotte et al., 2006). CRFs belong to the *AP2/ERF* super‐family of transcription factors and are present and highly conserved among all land plants (Rashotte & Geortzen, 2010; Zwack et al., 2012). Within the CRFs, there are five evolutionarily distinct clades, each with unique functional regulation (Hallmark & Rashotte, 2019). Cytokinin Response Factor 6 (CRF6) is one of two members of CRF clade III in Arabidopsis and may be the CRF gene best examined for stress‐response function. CRF6 is known to be CK induced, expressed in the vasculature, and has been shown to be involved in mitigation of oxidative stress, yet its role in the CK signal cascade is underexplored, particularly in relation to CK‐regulated downstream targets of this transcription factor (Hallmark & Rashotte, 2019; Rashotte et al., 2006; Zwack et al., 2012, 2016).

In order to determine downstream targets of CRF6 and potential roles that CRF6 plays in regulating CK response, the transcriptome of a knockout CRF6 mutant line (*crf6‐*2) responding to CK treatment was analyzed and compared to that of wildtype (WT) for mis‐regulated genes. This analysis revealed numerous CK‐regulated differentially expressed genes (DEGs) with altered regulation in a *crf6* mutant background. These DEGs were considered to be CK regulated in a CRF6‐dependent manner or CRF6 CK‐regulated targets. Among these CRF6 targets were genes involved in CK signaling, plant growth and development, and biosynthetic processes, as well as a subset identified as having potassium uptake and transport functions.

Although CK has been previously linked to physiological changes in plant growth resulting from potassium deficiency (Nam et al., 2012), the mechanisms and genes by connecting these processes have yet to be identified. Potassium (K+) is crucial for processes from plant‐wide growth to cellular functions where it has many critical roles, including ionic balance, neutralizing negative charges on proteins, maintaining cellular pH, and enzymatic activation (Beringer & Troldenier, 1980; Demidchik, 2014; Prajapati & Modi, 2012). In order to investigate the means by which CRF6 and CK are connected to potassium uptake, transportation, and deficiency, the CRF6‐dependent CK‐induced regulation of high‐affinity potassium transporter 5 (*HAK5*), stellar potassium outward rectifying transporter (*SKOR*), and nitrate transporter 1.5 (*NRT1.5*) were explored.

## 2 RESULTS

### 2.1 CK transcriptional response in WT and crf6 A. thaliana seedlings

To determine the transcriptional effects of CK on *A. thaliana,* 10‐day‐old seedlings were exogenously treated with CK (2 µM 6‐benzyl adenine or BA) or a control (0.2% DMSO) for 6 hr and changes in transcriptomic responses were analyzed using Affymetrix Arabidopsis ST 1.0 microarrays. Statistically significant DEGs were identified and fold change (FC) of transcription levels was calculated in response to CK. CK treatment of wildtype (WT), Col‐0, plants resulted in 580 DEGs that were transcriptionally altered in response to the hormone. Significant (*p*adj > .05) DEGs were found to be induced to 150% and repressed to 50% of their original (control) expression levels were selected and further analyzed for Gene Ontology (GO) terms using GO Enrichment Analysis (Table 1).

**Table 1:** GO term enrichment analysis of the Genes that were induced to at least 150% and repressed by at least half of their transcription in WT with the addition of CK. Fold enrichment (FE) of the terms and false discovery rate (FDR) are indicated

GO biological process complete	FE	FDR
de novo' IMP biosynthetic process	35.15	2.70E‐03
IMP biosynthetic process	26.36	4.91E‐03
Cytokinin catabolic process	26.36	3.09E‐02
IMP metabolic process	23.43	6.47E‐03
Purine ribonucleoside monophosphate biosynthetic process	23.07	3.48E‐05
Purine ribonucleoside monophosphate metabolic process	23.07	3.34E‐05
Purine nucleoside monophosphate biosynthetic process	23.07	3.21E‐05
Purine nucleoside monophosphate metabolic process	23.07	3.09E‐05
rRNA pseudouridine synthesis	22.6	3.98E‐02
Syncytium formation	19.77	3.56E‐04
Ribonucleoside monophosphate biosynthetic process	12.73	5.62E‐04
Cleavage involved in rRNA processing	12.65	2.42E‐03
Nucleoside monophosphate biosynthetic process	12.41	1.57E‐04
Ribonucleoside monophosphate metabolic process	12.3	6.51E‐04
Nucleoside monophosphate metabolic process	12.05	1.84E‐04
Maturation of LSU‐rRNA from tricistronic rRNA transcript	11.72	3.87E‐02
snRNA metabolic process	11.46	1.23E‐02
RNA phosphodiester bond hydrolysis, endonucleolytic	10.54	4.92E‐02
Maturation of SSU‐rRNA from tricistronic rRNA transcript	10.54	1.50E‐03
Maturation of SSU‐rRNA	10.14	4.23E‐05
Cytokinin‐activated signaling pathway	9.95	4.63E‐05
Cellular response to cytokinin stimulus	9.41	7.04E‐05
Ribosomal large subunit biogenesis	8.87	1.01E‐08
Cytokinin metabolic process	8.79	1.02E‐02
Purine ribonucleoside metabolic process	8.79	2.89E‐02
rRNA processing	8.71	1.12E‐19
Plant‐type cell wall loosening	8.55	1.13E‐02
rRNA metabolic process	8.5	1.88E‐19
Response to cytokinin	8.41	6.14E‐07
RNA phosphodiester bond hydrolysis	8.2	4.76E‐03
Ribosome biogenesis	8.11	2.66E‐26
Purine nucleoside metabolic process	7.99	3.89E‐02
Ribosomal small subunit biogenesis	7.97	1.18E‐05
Ribosomal large subunit assembly	7.36	2.05E‐02
Translational elongation	7.32	4.91E‐02
Cellular hormone metabolic process	7.24	8.46E‐03
Ribonucleoprotein complex biogenesis	7	9.36E‐25
Maturation of LSU‐rRNA	6.88	2.65E‐02
Ribosome assembly	6.82	2.84E‐04
ncRNA processing	6.28	2.70E‐16
Inorganic anion transport	5.98	7.65E‐04
Glycosyl compound biosynthetic process	5.86	2.26E‐02
ncRNA metabolic process	5.57	1.77E‐15
Nucleic acid phosphodiester bond hydrolysis	5.27	1.81E‐02
Cytoplasmic translation	5.2	3.76E‐02
Ribonucleotide biosynthetic process	5.17	4.78E‐03
Purine ribonucleotide biosynthetic process	5.02	2.28E‐02
Ribose phosphate biosynthetic process	4.93	6.10E‐03
Amine metabolic process	4.61	1.85E‐02
Purine‐containing compound biosynthetic process	4.56	1.95E‐02
Purine nucleotide biosynthetic process	4.54	3.68E‐02
Ribonucleoprotein complex assembly	4.45	2.07E‐03
Ribonucleoprotein complex subunit organization	4.37	2.39E‐03
Nucleotide biosynthetic process	4.33	4.64E‐03
Nucleoside phosphate biosynthetic process	4.27	4.65E‐03
Embryo sac development	4.03	1.29E‐02
Plant‐type cell wall organization	3.8	4.95E‐02
RNA processing	3.35	9.17E‐10
Organelle assembly	3.31	4.19E‐02
Plant‐type cell wall organization or biogenesis	3.15	1.86E‐02
Hormone metabolic process	3.05	2.36E‐02
Cellular component biogenesis	2.89	8.54E‐11
Gene expression	2.82	2.34E‐12
Translation	2.69	4.72E‐03
Peptide biosynthetic process	2.66	5.12E‐03
RNA metabolic process	2.62	1.15E‐08
Peptide metabolic process	2.55	3.76E‐03
Regulation of hormone levels	2.54	2.88E‐02
Nucleobase‐containing compound biosynthetic process	2.5	2.49E‐02
Cellular nitrogen compound biosynthetic process	2.37	3.55E‐05
Amide biosynthetic process	2.31	2.80E‐02
Cellular response to hormone stimulus	2.28	1.30E‐02
Response to osmotic stress	2.23	4.01E‐02
Cellular amide metabolic process	2.22	1.60E‐02
Cellular response to endogenous stimulus	2.21	1.61E‐02
Response to hormone	2.21	1.37E‐05
Heterocycle biosynthetic process	2.2	2.65E‐02
Response to endogenous stimulus	2.18	2.48E‐05
Nucleic acid metabolic process	2.17	2.31E‐06
Cellular nitrogen compound metabolic process	2.15	3.19E‐11
Heterocycle metabolic process	2.15	4.05E‐09
Nucleobase‐containing compound metabolic process	2.15	9.17E‐08
Cellular aromatic compound metabolic process	2.06	3.10E‐08
Aromatic compound biosynthetic process	2.03	4.93E‐02
Organic cyclic compound metabolic process	2.03	3.63E‐08
Cellular response to organic substance	2.03	3.79E‐02
Response to lipid	2.02	4.17E‐02
Organic cyclic compound biosynthetic process	2.01	3.89E‐02
Organonitrogen compound biosynthetic process	2.01	2.86E‐03
Response to organic substance	1.97	7.44E‐05
Response to acid chemical	1.97	4.94E‐03
Response to chemical	1.92	2.46E‐07
Response to inorganic substance	1.89	4.16E‐02
Response to oxygen‐containing compound	1.82	4.62E‐03
Cellular response to chemical stimulus	1.78	4.45E‐02
Cellular component organization or biogenesis	1.74	4.20E‐05
Cellular biosynthetic process	1.68	3.04E‐03
Organic substance biosynthetic process	1.65	4.57E‐03
Biosynthetic process	1.64	2.99E‐03
System development	1.6	3.98E‐02
Anatomical structure development	1.55	4.93E‐03
Developmental process	1.55	4.58E‐03
Multicellular organism development	1.54	1.79E‐02
Multicellular organismal process	1.5	2.24E‐02
Nitrogen compound metabolic process	1.43	5.54E‐04
Response to stimulus	1.41	2.05E‐03
Organic substance metabolic process	1.4	1.06E‐04
Metabolic process	1.4	1.54E‐05
Cellular metabolic process	1.38	2.87E‐04
Primary metabolic process	1.33	1.05E‐02
Cellular process	1.28	4.93E‐04
Protein modification process	0.47	3.53E‐02
Cellular protein modification process	0.47	3.49E‐02
Vesicle‐mediated transport	<0.01	3.02E‐02

There were 429 CK‐induced DEGs of which the most highly upregulated genes included several type‐A Arabidopsis Response Regulators (*ARRs*): *ARR3, ARR5, ARR6, ARR7, ARR15, and ARR16* (Table S1). Type‐A ARRs are well known as highly CK inducible and serve as negative regulators of CK response in plants. GO‐enrichment analysis of CK‐induced genes in the WT background treated samples included the enrichment terms “cytokinin signaling” and “hormone response” as expected (Table 1). There were 151 CK‐repressed genes, including the most highly repressed genes: *HAK5*, *NRT2.1*, connected to ion transport and reduction and in growth regulation (Table 1, Table S1). Overall, these findings are consistent with previous transcriptome analysis of CK‐treated Arabidopsis and show a standard transcriptional response to CK occurred in this study (Table S1; Bhargava et al., 2013).

### 2.2 CRF6 dependently CK‐regulated genes

**FIGURE 1:**
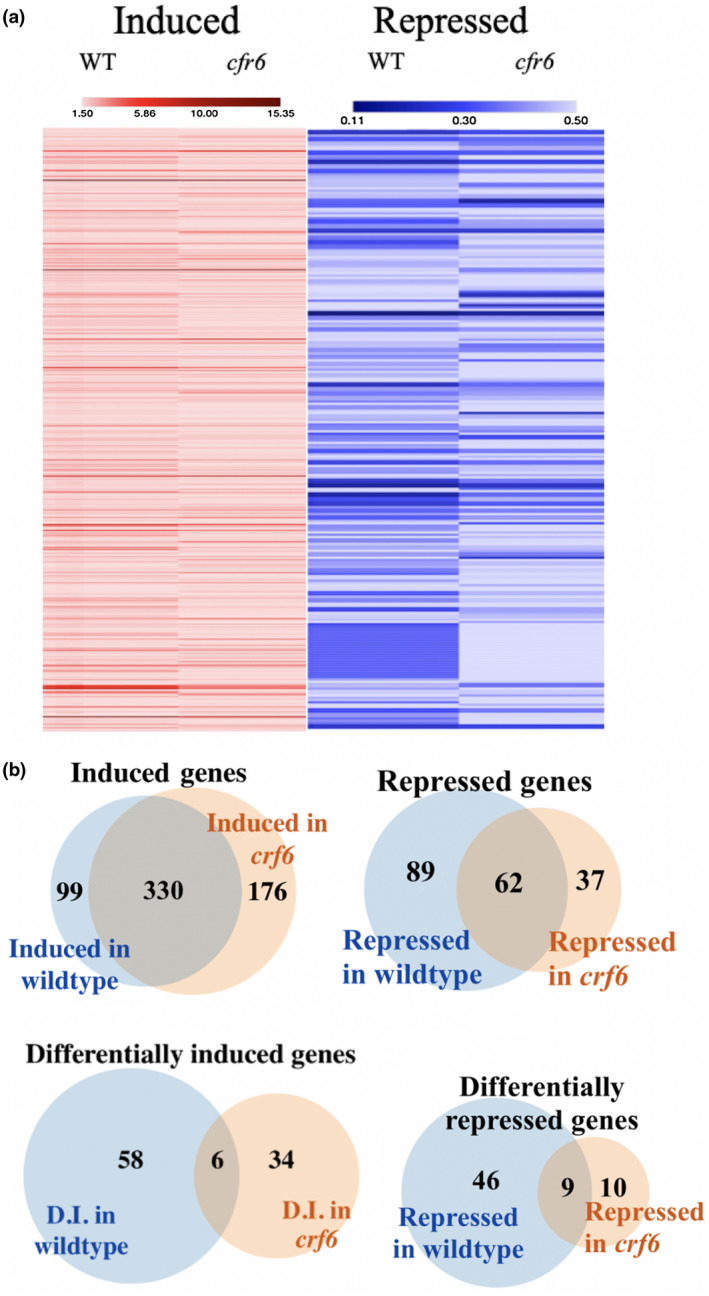
(a) Heat map depicting the fold change in expression of genes in WT and *crf6* mutant plants when treated and untreated with CK and separated into induced and repressed categories. (b) Venn diagrams illustrating the crossover between the genes between genes that were induced (addition of CK caused 1.5 times greater transcription of the gene) and were repressed (addition of CK caused transcription of the gene to be reduced to half or lower) and the genes that were differentially altered in transcription in the *crf6* mutant plants

In order to determine genes that are regulated in a CK‐dependent manner by the transcription factor CRF6, a genome‐wide transcriptome analysis of *crf6‐2*, a knockout line confirmed in Zwack et al., 2012, and WT seedlings treated for 6 hr with CK (2 µM BA) or with 0.2% DMSO. Transcriptomes were analyzed using Affymetrix Arabidopsis ST 1.0 microarrays. Differences in gene expression between the WT and *crf6‐2* mutant with and without exogenous CK treatment are depicted in heat maps of fold change (FC) differences (Figure 1; Table S2). As noted above, CK treatment of WT revealed a total of 579 significant DEGs in CK‐treated versus control plants: 429 induced and 151 repressed. CK treatment of *crf6* resulted in 163 significantly differentially regulated genes versus control treatment: 98 induced and 65 repressed (Figure 1). Genes that are induced or repressed after CK treatment to a greater or lesser degree (a difference in regulation of at least 50%) in the mutant background, as compared to the regulation observed in WT were determined to be CRF6 dependantly regulated genes (CDRGs). Of the 98 genes exhibiting at least a 50% difference in CK induction in *crf6* versus WT, 64 CDRGs were found to be part of 428 CK‐induced DEGs of WT (Figure 1). Whereas, of the 65 genes that were at least 50% differentially repressed in *crf6* background, 55 CDRGs were found to be part of the 151 CK‐repressed DEGs of WT (Figure 1). These findings indicate that CRF6 is required for a subset of WT‐CK gene regulation (~15% of the induced WT DEGs, 64 of 429, and ~36% of the repressed WT DEGs, 55 of 151). Additionally, this indicates that CRF6 is required for hindering the non‐standard CK regulation of a subset of genes in WT plants (34 induced CDRGs and 10 repressed). Since, CRF6 appears to be responsible for a larger regulation of CK‐repressed CDRGs, we focused additional examinations on this gene set. An examination of CRF6‐dependent CK‐repressed CDRGs for GO term enrichment analysis (Table 2) revealed several terms related to ion transport. A more detailed examination of these metal and ion transport‐related genes as seen in Table 3 revealed that three of the top four CDRGs in this group were involved as having functions related to potassium (K+) uptake or transport; high‐affinity potassium transporter 5 (*HAK5*), stellar potassium outward rectifying transporter (*SKOR*), and nitrate transporter 1.5 (*NRT1.5*). To investigate the potential relationships among potassium, CK, and CRF6, these genes were selected for further investigation.

**Table 2:** The GO analysis of term enrichment in genes that were differentially regulated in the *crf6* plants when treated with CK as compared to wildtype

Upregulated		
GO biological process complete	FE	FDR
Thalianol metabolic process (GO: 0080003)	> 100	3.01E‐03
Tricyclic triterpenoid metabolic process (GO: 0010683)	> 100	2.63E‐03
Downregulated		
GO biological process complete	FE	FDR
Inorganic cation transmembrane transport (GO: 0098662)	13.2	3.19E‐02
Cation transmembrane transport (GO: 0098655)	12.38	3.13E‐02
Inorganic ion transmembrane transport (GO: 0098660)	12.03	3.27E‐02
Innate immune response (GO: 0045087)	10.35	3.19E‐02
Immune response (GO: 0006955)	10.21	2.86E‐02
Ion transmembrane transport (GO: 0034220)	9.35	3.09E‐02
Immune system process (GO: 0002376)	8.34	3.68E‐02
Response to bacterium (GO: 0009617)	7.59	3.27E‐02
Response to external biotic stimulus (GO: 0043207)	5.57	2.12E‐02
Response to other organism (GO: 0051707)	5.57	1.06E‐02
Response to biotic stimulus (GO: 0009607)	5.57	7.13E‐03
Transmembrane transport (GO: 0055085)	5.55	3.62E‐02
Defense response to other organism (GO: 0098542)	5.42	4.03E‐02
Response to acid chemical (GO:0001101)	4.59	3.21E‐02
Multi‐organism process (GO: 0051704)	4.15	3.45E‐02
Response to external stimulus (GO: 0009605)	4	3.48E‐02
Response to oxygen‐containing compound (GO: 1901700)	3.78	4.31E‐02
GO cellular component complete		
Extracellular region (GO: 0005576)	2.85	1.24E‐02
Cell periphery (GO: 0071944)	2.62	7.57E‐03

**Table 3:** Metal‐ and ion‐related DEGs in WT and *crf6* in response to CK. Fold change in expression of the DEGs identified as metal and ion transport‐related from GO enrichment analysis are noted as fully described in Table S1 and Table S2. Delta value for each gene is the FC *crf6* vs. *crf6+*CK value divided by the FC WT vs. WT+CK values. Genes labeled as “increased” and “reduced” are alternatively regulated

At#	Gene Description	Symbol	Predicted Gene Function	FC Col vs. Col+CK	padj	FC *crf6* vs. *crf6+CK*	padj	Delta *crf6* to Col CK Treatment Regulation	
AT5G09690	Magnesium Transporter 7	MGT7	Magnesium transport	0.4487	0.006995	0.9287	0.90282	2.0700	Increased
AT4G13420	Affinity K+ Transporter 5	HAK5	K+ uptake	0.1670	1.67E‐05	0.3398	0.01742	2.0353	Increased
AT3G02850	Stelar K+ Outward Rectifier	SKOR	K+ transport/homeostasis	0.3872	0.032512	0.6207	0.13213	1.6029	Increased
AT1G32450	Nitrate Transporter 1.5	NRT1.5	K+ transport/homeostasis, Nitrate transport	0.4822	0.004754	0.7577	0.08566	1.5713	Increased
AT1G01580	Ferric Reduction Oxidase 2	FRO2	Iron uptake	0.2160	0.000411	0.2742	0.00166	1.2691	Similar
AT2G30766	Fe‐Uptake‐Inducing Peptide1	FEP1	Iron uptake regulator	1.6023	0.76095	2.0110	0.00545	1.2551	Similar
AT4G19690	Iron‐Regulated Transporter 1	IRT1	Iron homeostasis	0.3255	0.004095	0.4083	0.01516	1.2545	Similar
AT3G12750	Zinc Transporter 1 Precursor	ZIP1	Zinc transport	0.4139	0.002764	0.5082	0.01088	1.2277	Similar
AT4G27860	Membrane of ER Body 1	MEB1	Manganese homeostasis, Intracellular iron sequestering	1.3726	0.22509	1.5344	0.00323	1.1178	Similar
AT4G34600	Casparian Strip Integrity Factor 2	CIF2	Iron homeostasis	0.4165	0.009451	0.4599	0.00212	1.1043	Similar
AT5G24580	Heavy Metal Associated Protein 47	HMP47	Metal ion transport	1.7219	0.16163	1.8898	0.01047	1.0975	Similar
AT2G28160	Arabidopsis Fe‐Deficiency‐Induced Transcription Factor 1	FIT1	Iron uptake regulator	0.3868	0.00663	0.4121	0.00043	1.0653	Similar
AT3G59820	Leucine Zipper‐EF‐Hand‐Containing Transmembrane Protein 1	LETM1	Metal ion homeostasis	1.6059	0.05732	1.5005	0.01275	0.9344	Similar
AT1G58340	Arabidopsis Abnormal Shoot4	ABS4	Iron homeostasis	3.4829	0.00052	2.4905	0.00244	0.7151	Similar
AT3G63380	Auto‐Inhibited Ca2 + Atpase 12	ACA12	Calcium transport	0.6466	0.42115	0.4436	0.01875	0.6862	Reduced
AT2G24720	Glutamate Receptor 2.2	GLR2.2	Calcium homeostasis	1.0045	0.99762	0.4180	0.00393	0.4161	Reduced

In order to ensure that transcriptome findings and this CRF6‐CK‐postassium connection was valid before further examination, qRT‐PCR expression analysis was conducted. RNA of *crf6* and WT seedlings was extracted in new biological replicates treated as they had been for the microarray analysis (Table 4). qRT‐PCR expression analysis indicates a significant reduction in FC between the WT, and *crf6* in expression of the three K+‐related genes, confirming general microarray expression findings and that their CK repression is CRF6 dependent.

**Table 4:** Verification of CRF6 CK‐regulated targets. The fold change difference in expression of HAK5, NRT1.5, and SKOR in WT and *crf6* in the presence of CK (+) 2 µM BA or absence of CK (Control) as conducted for transcriptome analysis, as determined via qRT‐PCR. Presented as an Average ± Standard Deviation. All data from microarray have an adjusted *p* < .05

Gene	qRT‐PCR		Microarray	
	WT control vs. WT+CK	*crf6* control vs. *crf6*+CK	WT control vs. WT+CK	*crf6* control vs. *crf6*+CK
HAK5	−4.64 ± 1.26	−0.23 ± 1.24	−5.99	−2.94
NRT1.5	−5.08 ± 1.87	−0.83 ± 1.69	−2.07	−1.32
SKOR	−3.19 ± 2.10	−0.54 ± 1.51	−2.58	−1.61

### 2.3 Physiological examinations of CRF6‐dependent CK–potassium interactions

In order to determine if CRF6 is involved in CK‐regulated potassium interactions, several mutant lines were examined, to observe the response of K+ mutants to altered CK treatment and CRF6 to altered K+ content. The three potential CRF6‐dependent CK‐regulated target genes function in different aspects of potassium movement: both SKOR and NRT1.5 are transporters involved in loading K+ into the root xylem, allowing for movement of K+ to aerial tissues, and HAK5 is a K+ transporter involved in facilitating K+ into the root in K+‐deficient environments (Ahn et al., 2004; Gierth et al., 2005; Park et al., 2008; Rubio et al., 2008; Qi et al., 2008). Along with mutants of these three K+‐related lines, two *CRF6* altered lines (the mutant knockout *crf6‐2* and overexpressor *CRF6OE,* previously described in Zwack et al., 2012, 2016) were examined.

Seedlings 4 days after germination (DAG) were transferred to one of four types of media to grow for 10 days. These Murashige and Skoog(MS)‐based media types contained the standard K+ levels of MS media (Standard K), or reduced K+ levels (Low K), and each K+ media either did not contain or contained (+ CK) CK as detailed in Material and Methods. Measurements were taken of maximum quantum yield of photosystem II, or Fv/Fm, as well as growth of root and shoot tissues separately in order to examine changes related to K+ and CK. Under standard growth conditions mutant and altered lines showed only minor physiological or growth differences from WT plants, but significant differences were found within treatment groups.

### 2.4 Fv/Fm response to cytokinin and low K^+^

**FIGURE 2:**
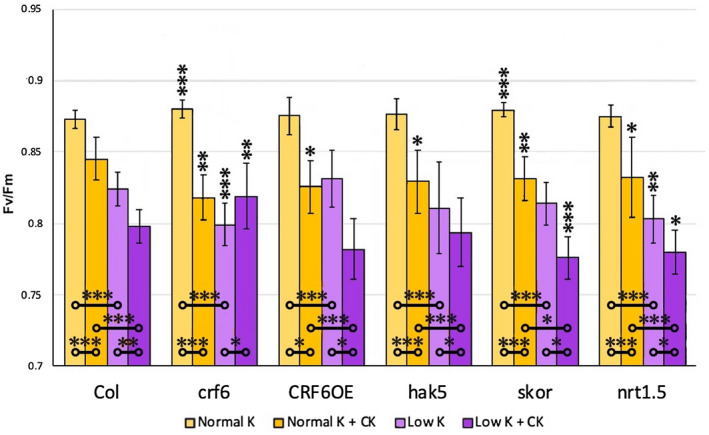
FV/FM measurements were taken to gauge the overall health of the plants after 10 days. Asterisks on comparison bars indicate significance level; *p ≤ .05, **p ≤ .005, ***p ≤ .0005. Comparison bars between treatments within a line are indicated closer to the x axis with each end indicating the compared data. The asterisks above the bars indicate the significance level of the data as compared to the same treatment in WT

In order to determine if there were connections among CRF6, CK response, and potassium, with photosynthetic efficiency (as an indicator of plant fitness), measurements of maximum quantum yield of photosystem II or Fv/Fm was conducted in distinct mutant lines and under altered growing conditions (Figure 2). Specifically, Fv/Fm was measured in seedlings that were grown for 10 days on MS media with normal or low K+ levels ± CK under standard growth conditions. The highest Fv/Fm levels for all genotypes were found when grown on standard media without CK (Figure 2). While only small changes in Fv/Fm levels found from these examinations, even minor differences in photosystem II maximum quantum yield are well‐known to be of great importance to photosynthetic performance.

Interestingly, although *crf6* shows significantly, albeit slightly higher Fv/Fm levels compared to WT on standard media to WT, the converse was found when grown on CK‐supplemented media (Figure 2). *crf6* showed a stronger reduction in Fv/Fm (7% compared to 3.2% in WT) when compared to non‐CK supplemented media. These changes in response to CK are consistent with CRF6 playing a role in CK‐regulated processes such as photosynthesis. Moreover, an examination of *crf6* under low versus standard K^+^ conditions revealed a much larger reduction in Fv/Fm levels than WT (9.2% in *crf6* and 5.6% in WT: Figure 2). However, unlike WT where Fv/Fm levels were reduced by 3.2%, *crf6* demonstrated a slight but significant recovery of about 2.5% when CK was present in the low K^+^ media (Figure 2).

The *CRF6OE* line reacted very differently to the growing conditions than either the WT or *crf6* line. On standard K^+^ media, in the presence of CK, the *CRF6OE* line exhibited a greater reduction in Fv/Fm levels (5.6% compared to 3.2% in WT: Figure 2). Unlike the *crf6* line, when CK was added to the low K^+^ media, there was no recovery in photosynthetic response.

When CK was added to the standard media, each of the three potassium‐related transporter mutant lines showed small but significant reductions in Fv/Fm levels as compared to those of the WT (*hak5* 1.9%, *skor* 1.6%*, nrt1.5* 1.6%: Figure 2).

The *skor* mutant exhibited a different pattern of Fv/Fm levels across growing conditions. This includes the minor reduction (1.6%) in when CK was added to standard media, yet distinctly when CK was added to the low potassium media, there was a 4.7% reduction compared to that of the WT (Figure 2).

The *nrt1.5* mutant line had Fv/Fm levels similar to WT when grown on standard media. On all other media there was a significantly greater reduction in Fv/Fm levels than observed in WT. *nrt1.5* plants grown on standard media with CK were reduced in Fv/Fm by 1.6%, those grown on low potassium by 1.2%, and those on low potassium with CK by 2.7% (Figure 2). Overall, these findings suggest that there are connections between CK and potassium that appear to be CRF6 related, as measured by examination of photosynthetic response.

### 2.5 Root growth in response to CK and low K^+^

**FIGURE 3:**
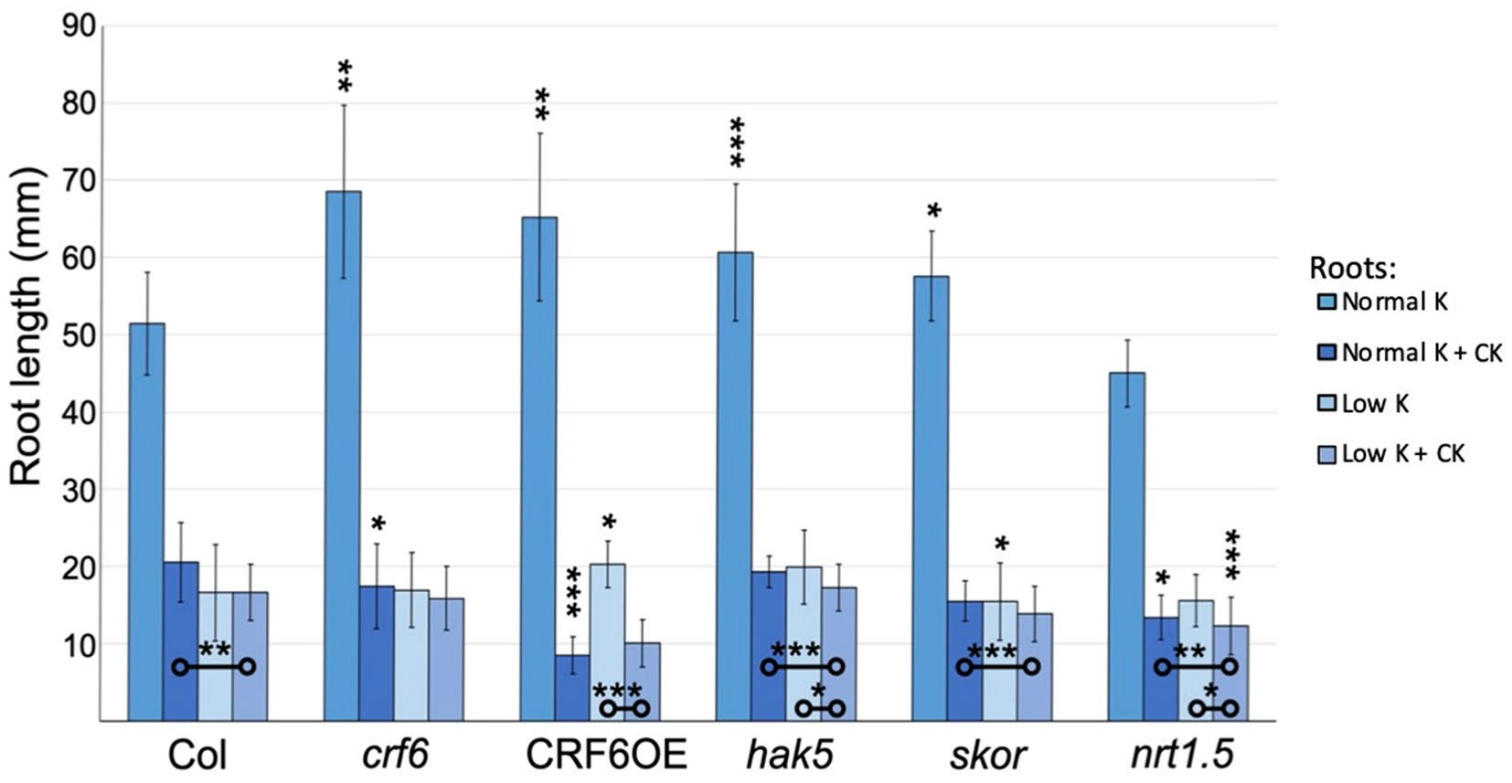
Root growth, in millimeters, 10 days after transfer to treatment and control plates. Asterisks indicate significance level; *p ≤ .05, **p ≤ .005, ***p ≤ .0005 when an ANOVA Ad Hoc test was used. Asterisks above the error bars indicate significance of the length of the roots of that line as compared to that of the wildtype grown in the same treatment. The asterisks on comparison bars closer to the x axis indicate significance of differences within lines between different treatments. All plant roots grown on standard potassium media were significantly different from those grown on treatment plates (*p* < .0005)

Root growth as a measure of overall plant health was assessed in seedlings grown on media with different CK and K^+^ levels, as conducted for Fv/Fm analyses. Once germinated on standard media, 4‐day‐old seedlings were transferred to different treatment media and after 10 days primary root growth was measured (Figure 3). On standard MS media without CK, *nrt1.5* and WT root growth was similar, while *crf6, skor,* and *hak5* mutant lines as well as the *CRF6OE* line had significantly longer roots (Figure 3). Roots were 33.2%, 26.8%, 38.4%, and 11.9% longer for *crf6, CRF6OE*, *hak5*, and *skor*, respectively, than those of WT. CK treatment generally resulted in a strong reduction in root growth for all genotypes. Despite the greater growth on standard media of some lines, there was no increase in root length versus WT for any lines when CK was present in the media. Root growth of both *crf6* and *CRF6OE* lines was more strongly reduced when grown on the standard media plus CK than was that of the WT line. *crf6* root growth was slightly, although significantly more affected (85.9% of the growth than observed in WT), whereas roots of the *CRF6OE* line grew only 41.2% of the length of WT (Figure 3). As observed in *crf6*, root growth in the *nrt1.5* line was slightly more strongly affected (growing 9.8% less) than that of the WT line.

When all lines were grown on low K+ media, only the root growth of *CRF6OE* and *skor* plants differed significantly from that of WT. *CRF6OE* showed 22.2% longer root growth than observed in WT, while that of *skor* was 6.9% shorter (Figure 3).

Roots of WT*, crf6,* and *skor* lines grown on low K+ media in the presence of CK did not grow significantly shorter than they had on the same media without CK (Figure 3), but *CRF6OE*, *hak5*, and *nrt1.5* plants did show diminished root growth when CK was added to the low potassium media (75.1% and 74.0% of their growth without CK, respectively). When CK was present in the low K+ media, the increased root growth observed in *CRF6OE* on standard media without CK was negated. Roots of the *CRF6OE* line grew only 60.1% and 44.1% of the total length of the WT growing on low potassium media with and without CK, respectively. Root growth of *CRF6OE* plants was not significantly different from WT when grown on either media containing CK.

### 2.6 Rosette diameter in response to CK and low K^+^

**FIGURE 4:**
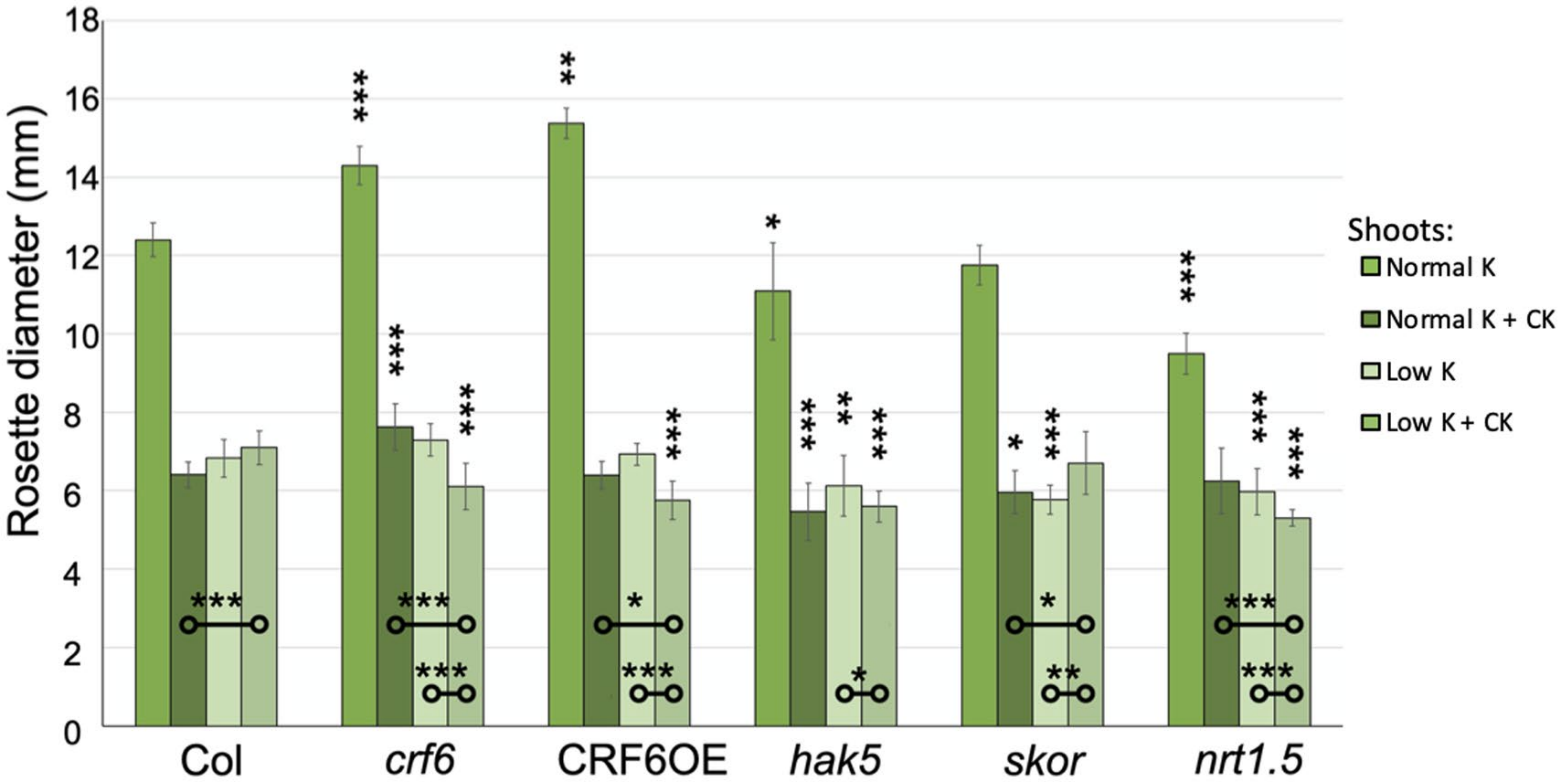
Rosette diameter, in millimeters, 10 days after transfer to treatment and control plates. Asterisks indicate significance level; *p ≤ .05, **p ≤ .005, ***p ≤ .0005, when an ANOVA Ad Hoc test was used. Asterisks above the error bars indicate significance of the length of the roots of that line as compared to that of the wildtype grown in the same treatment. The asterisks on comparison bars closer to the x axis indicate significance of differences within lines between different treatments. All plant rosettes grown on standard potassium media were significantly wider than those grown on treatment plates (*p* < .0005)

Above‐ground plant size as measured by rosette diameter (shoot growth) was conducted when seedlings were 14 days old. The shoot growth of WT, *CRF6OE*, *crf6, hak5, skor,* and *nrt1.5* exposed to the same set of CK and K+ media treatments showed a different pattern than observed in roots (Figures 3 and 4). On standard media, *crf6* and *CRF6OE* had larger shoots than those of WT (by 15.3% and 24.0%, respectively), while the *hak5* and *nrt1.5* seedlings had smaller shoots (by 5.2% and 23.4% of that of WT) (Figure 4).

With the addition of CK to the standard media, *crf6* seedlings grew to have 19.1% larger rosettes than WT, while both *hak5* and *skor* had smaller rosettes (by 14.7% and 6.9%, respectively). The *CRF6OE* and *nrt1.5* lines, which had been significantly different in size from the WT on standard media, exhibited growth similar to that of WT when CK was added (Figure 4).

When the plants were grown on low K+ media, both altered CRF6 lines exhibited growth similar to that of WT, while all K+‐related lines all grew smaller shoots. *hak5, skor,* and *nrt1.5* grew to be 10.3%, 15.5%, and 12.5% of the size of the rosettes of WT, respectively.

When CK was added to the low K+ media, the lines exhibited different levels of growth. The *crf6*, *CRF6OE*, *hak5,* and *nrt1.5* lines all grew significantly smaller shoots than did WT, by 13.9%, 19.0%, 21.1%, and 25.2%. The *skor* line did not differ from WT; although the low K+ media without CK did grow smaller than did WT, the addition of CK resulted in larger rosettes in the *skor* line and phenotypic rescue (Figure 4).

Most genotypes grew smaller shoots on low K+ media with CK compared to standard K+ media with CK (Figure 4). However, the shoots of both the WT and *skor* seedlings grew larger rosettes on the low K+ media with CK than they had on the standard K+ media with CK. WT and *skor* exhibited an increase in growth by 10.8% and 12.5% on low K+ media with CK, while *crf6, CRF6OE*, and *nrt1.5* grew rosettes that were 19.9%, 10.1%, and 15.1% smaller than they had been when they were grown on standard K+ media with CK.

### 2.7 Measurements of plant potassium levels in response to CK and low K^+^ conditions

**FIGURE 5:**
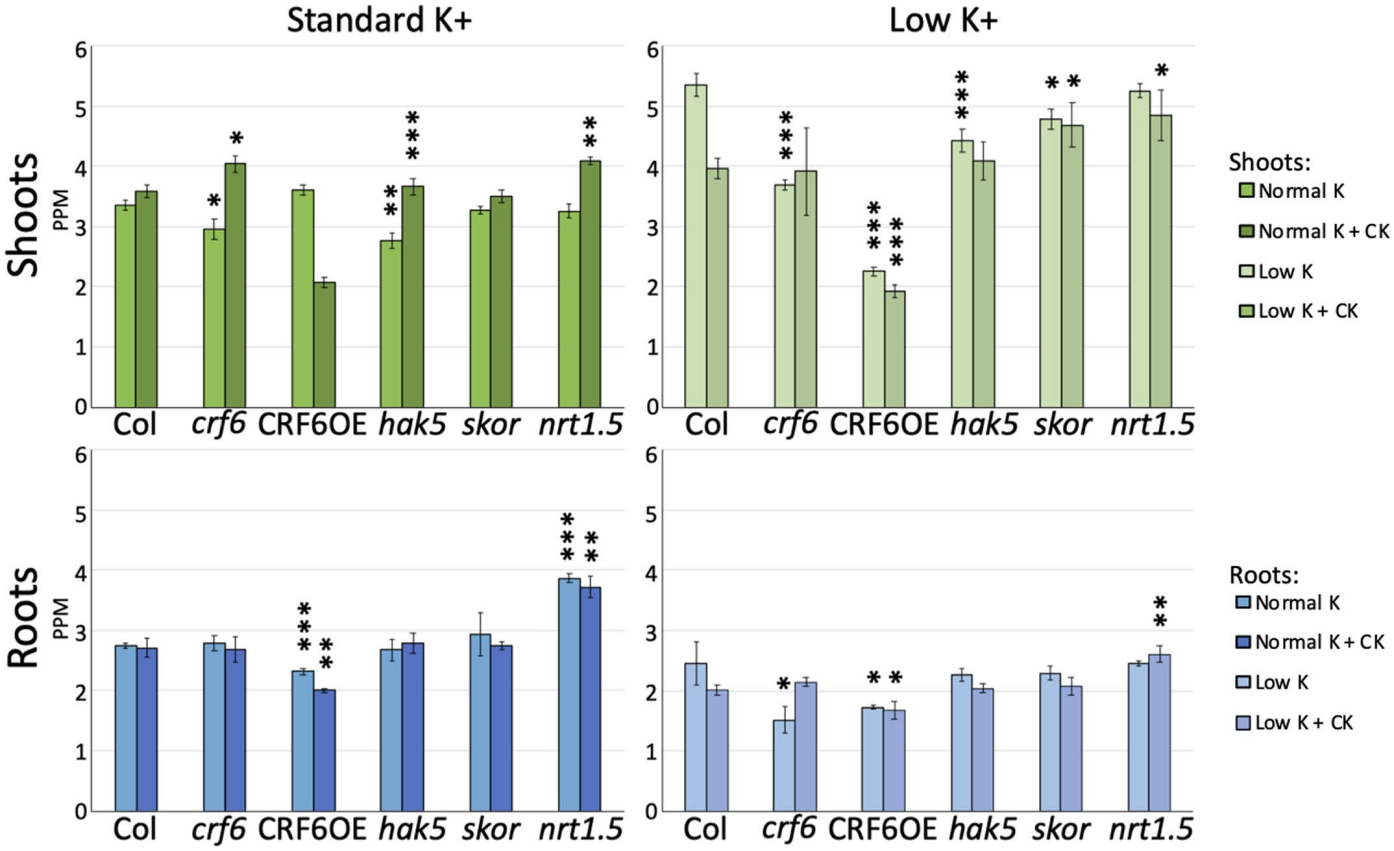
Potassium levels as measured by ICP‐OES collected from A. shoots and B. roots. Asterisks indicate significance level; *p ≤ .05, **p ≤ .005, ***p ≤ .0005 when an Ad Hoc test was used to compare that line, treatment, and tissue type to the WT of the same treatment and tissue type

To further investigate the potential relationship among CK, CRF6, and potassium movement, the K+ content of the roots and shoots of WT, *crf6*, *CRF6OE*, *hak5, skor,* and *nrt1.5* was analyzed after plants were grown on media containing normal and reduced K+ levels in the presence and absence of cytokinin as done in other analyses (Figure 5).

#### 2.7.1 K^+^ concentration in aerial tissue (shoots)

In order to better understand if K+ levels were altered in response to CK, seedling tissue (split into shoots and roots) of CRF6 altered lines and the K+ transporting mutants were examined using ICP‐OES analysis to measure free ion levels (Figure 5; Table 5). When seedlings were grown on standard media, the K+ concentration of the aerial or shoot tissue varied from that of the WT in both the *crf6* and *hak5* (both were reduced, to 88.5% and 82.4%, respectively, of wildtype) (Figure 5). The K+ content of the *CRF6OE*, *skor*, and *nrt1.5* shoots did not differ significantly from that of WT.

**Table 5:** Additional comparisons made of the ionomics data shown in Figure 5 using ANOVA Post Hoc tests. The compared treatments are labeled along the top, and the line and tissue types are listed in the first two columns. Asterisks indicate significance level; *p ≤ .05, **p ≤ .005, ***p ≤ .0005

	Standard K+ vs. Standard K+ + CK	Standard K+ vs. Low K+	Low K+ vs Low K+ + CK	Standard K+ + CK Low K+ + CK
Shoots				
WT	*	***	***	*
crf6	**	**		
CRF6OE	***	***	*	
hak5	**	***		
skor	*	***		*
nrt1.5	***	***		*
Roots				
WT				**
crf6		**	*	*
CRF6OE	**	***		*
hak5		*		**
skor				**
nrt1.5		***		**

When CK was added to the standard media, the shoot tissue of *crf6, hak5,* and *nrt1.5* had higher concentrations of K+ compared to WT (112.6%, 102.1%, and 113.9% of WT, respectively). Only *skor* shoot tissue did not show significant differences versus WT in K+ concentration. The *CRF6OE* shoots were greatly reduced in K+ concentration, containing only 57.7% of the K+ present in WT.

The K+ concentration of WT shoots with CK added to standard media increased to 107.1% of that measured without the hormone. The *crf6, hak5,* and *nrt1.5* lines, which had shown higher K+ levels versus WT on standard media with CK, increased in shoot K+ concentration to 136.4%, 132.7%, and 125.4%, respectively, compared to K+ concentrations without CK (Figure 5). On standard media with CK, *CRF6OE* shoots were reduced to 57.4% of the K+ concentration of that measured without CK.

The levels of K+ present in the shoots differed when seedlings were grown on reduced K+ media (Figure 5). Shoot tissue of the *crf6*, *CRF6OE*, *hak5,* and *skor* lines contained lower K+ concentration than was observed in WT (68.9%, 42.2%, 82.7%, and 89.4% of the WT K+ concentration, respectively). When grown on low K+ media, only the K+ concentration of *nrt1.5* seedlings did not differ significantly from that of WT.

The K+ concentration in WT shoots increased to 159.7% of that observed on standard media when the seedlings were grown on low K+ media (Figure 5). Shoots of *nrt1.5* increased in K+ concentration by 161.3% when grown on low K+ media compared to that measured on standard media. The *crf6*, *hak5,* and *skor* shoots had increased in K+ concentration when grown on low K+ media, but not to the degree observed in WT (measuring 124.5%, 160.4%, and 146.7%, respectively, of their K+ concentration when grown on standard media). The *CRF6OE* line alone showed a reduction in shoot K+ concentration when grown on reduced K+ media (57.4% of that measured when the plants grew on standard media).

The concentration of K+ in shoot tissues shifted when CK was added to the low K+ media. Compared to that of WT, *skor* and *nrt1.5* showed greater concentrations of K+ (118.1% and 122.0%, respectively) of that in WT shoots, while the shoots of *CRF6OE* contained 48.6% of the K+ concentration measured in the WT. Neither the *crf6* nor the *hak5* shoot tissues differed significantly in K+ concentrations from that of WT when the seedlings were grown on reduced K+ media with CK.

The addition of CK to the reduced K+ media caused significant changes in K+ levels in shoot tissues of both WT and *CRF6OE* seedlings. The presence of CK in low K+ media yielded reduced K+ concentrations in shoots of WT and *CRF6OE* (to 74.1% and 85.4% of the concentrations observed on low K+ media without CK).

K+ concentrations of shoot tissues were affected differently when the plants were grown in the presence of CK on standard versus low K+ media (Figure 5). The WT, *skor,* and *nrt1.5* lines all showed a greater concentration of K+ in the shoot when grown on low K+ media with CK, 110.6%, 134.0%, and 118.7%, respectively, as compared to concentrations of those grown on standard media with CK. The K+ concentrations of *crf6*, *CRF6OE*, and *hak5* seedlings were not significantly affected.

#### 2.7.2 K+ concentration in root tissue

The K+ concentrations measured by ICP‐OES of roots collected with the shoot samples varied when grown on standard media (Figure 5; Table 5). Roots of *CRF6OE* and *nrt1.5* contained K+ at 84.2%, and 140.6% of the concentration observed in WT, respectively. K+ concentrations in the roots of the other lines were not significantly affected.

The *CRF6OE* and *nrt1.5* roots, alone, showed significant differences in K + levels versus WT when CK was added to the standard media, as they had when grown on standard media without CK (Figure 5). The *CRF6OE* roots contained 73.8% of the K + concentration measured in the WT roots, while that of *nrt1.5* measured 137.4% of WT.

Only in *CRF6OE* did the addition of CK to the standard media result in a significant change in root K + concentration. The addition of CK to the standard media caused the K + level in *CRF6OE* roots to decrease to 86.4% of that measured when grown on media without the hormone (Figure 5).

The K + concentration in the roots of *crf6* and *CRF6OE* differed significantly from that of WT when grown on reduced K + media. Roots of *crf6* and *CRF6OE* contained 61.7% and 70.1%, respectively, of the K + in WT roots (Figure 5).

When the plants were grown on low K + media, the K + concentration of the roots, compared to that measured on standard media, was reduced in all lines except WT and *skor* seedlings, which did not show a significant change in root K + concentration. In the roots of *crf6, CRF6OE*, *hak5,* and *nrt1.5*, K + root concentrations were 54.2%, 74.2%, 84.3%, and 63.5%, respectively, of those observed when the seedlings grew on standard media (Figure 5).

K + concentration of the roots of WT seedlings grown on low K + media did not differ significantly from that of WT seedlings grown on low K + media with added CK. The K + concentration in the roots of *CRF6OE* and *nrt1.5* diverged from that of WT; K + concentration in *CRF6OE* roots was 82.7%, and in *nrt1.5* roots was 129.4% of that measured in WT.

The addition of CK caused a significant change in the K + concentration of *crf6* roots. The K + concentration of *crf6* roots grown on reduced K + media with CK was 141.5% of the concentration of seedlings grown on low K + media without the CK. As observed in the shoots, the addition of CK to the low K + media resulted in a return to WT‐equivalent K + concentrations.

Root K + concentrations of seedlings grown on either media containing CK differed in all lines. In all lines grown on low K + media with CK, the K + concentration was reduced compared to that of those grown on standard media with CK. The concentration of K + in the roots of plants grown on low K + media with CK was 74.3% in WT, 79.7% in *crf6*, 83.3% in *CRF6OE*, 73.3% in *hak5*, 75.7% in *skor*, and 70.1% in *nrt1.5* of the concentration measured in roots of the same lines grown on standard media with CK.

## 3 DISCUSSION

Cytokinin Response Factors (CRFs) are known to be important AP2/ERF transcription factors connected to CK signaling and involved in the regulation of different abiotic stress responses (Hallmark & Rashotte, 2019; Rashotte et al., 2006). CRF6 has been previously examined for roles in senescence and oxidative stress response (Zwack et al., 2013, 2016), yet surprisingly there has been less examination of the role of CRF6 in CK response. Here, we used a subtractive transcriptomic approach to identify potential CK‐regulated targets of CRF6 in Arabidopsis. CK treatment (2 µM BA for 6 hr) of seedlings (10d) with and without CRF6 (WT and *crf6*) followed by genome‐wide transcript analysis revealed numerous CK‐regulated genes. Analysis of CK treatment in WT revealed 580 significantly Differentially Expressed Genes (DEGs) with at least a 50% change in the transcription levels (Figure 1; Table S1). Importantly, and as expected, many (*n* = 108) of those DEGs are well‐known CK‐regulated genes, appearing on the CK “golden list” of genes, defined by Bhargava et al., 2013, including some of the most typical genes; several type‐A Response Regulators (ARRs, negative regulators of CK response) and cytokinin oxidases (CKXs), which can also be noted from several cytokinin‐related terms identified in the GO enrichment analysis (Table 1). A similar examination for CK‐altered DEGs in the *crf6* background compared to those observed in WT revealed genes that require CRF6 for CK‐regulated activity.

Roughly, a 35% (55 of the 151) of WT CK‐repressed genes and 15% (64 of the 428) of WT CK‐induced genes were found to be altered in transcription by at least 50% when CRF6 was absent (Figure 1). This indicates that CRF6 is responsible for about 20% of the standard transcriptional response to CK (36.4% of repressed genes and 15.0% of induced genes) under the conditions examined. The bias toward affecting the CK‐repressed genes, suggests that CRF6 primarily functions as a negative regulator of cytokinin response, at least at this point in development.

The upregulated DEGs are involved in metabolic processes, and the repressed DEGs function primarily in ion transport and immune responses to both biotic and abiotic stimuli, including responses to oxygen species (Table 1). In Zwack et al. (2013, 2016), it was found that CRF6 may also be involved in regulation of a small subset of oxidative stress responses (~15%). While there is only minor overlap between the lists of the CRF6‐dependent DEGs relating to oxidative stress and CK treatment, there were specific CK‐related DEGs found as regulated by oxidative stress (Tables 1 and 2). These same genes do not appear to be connected to non‐oxidative stress CRF6‐CK signaling roles of CRF6, as they were not found to be similarly regulated with CK treatment. This could suggest that there are different functional roles for CRF6 even in a CK‐based manner under stress conditions.

As a major theme from GO‐enrichment terms of CRF6‐CK‐regulated DEGs was ion transport (Tables 2 and 5), we examined several of the specific DEGs that function with distinct roles in potassium transport: *HAK5*, *SKOR*, and *NRT1.5* (Table 2). *HAK5* is a cell membrane localized potassium channel that acquires K + from the external environment in K+‐limited conditions (Ahn et al., 2004; Gierth et al., 2005; Rubio et al., 2008). *SKOR* and *NRT1.5* are K + efflux transporters that release potassium into the apoplasm of the stele, allowing for the ion to be transported to the aerial tissues (Park et al., 2008). *SKOR* is the primary driver of K + loading into the xylem when a plant is in an environment with low K + content (Drechsler et at, 2015; Gaymard et al., 1998). *NRT1.5* (also known as AtNPF7.3) is a K+/H+‐coupled antiporter and, like *SKOR*, is expressed in the root pericycle and localized to the cell membrane (Drechsler et al., 2015; Li et al., 2017). As there has been little examination of CK and potassium transport, we decided to further investigate this connection and how CRF6 was involved in any potential regulation. This was conducted in by examining knockout mutants of *HAK5*, *SKOR*, and *NRT1.5*, which lack their respective gene expression and should roughly mimic the effects of cytokinin treatment in a WT background as seen from transcriptomic results.

We found that growth was affected for potassium mutants by exogenous addition of CK; the rosettes of the mutants grew smaller than those of the WT in the presence of CK in both types of media (Figure 4). This establishes a potassium–CK link. Similar effects in CRF6 altered backgrounds indicate that CRF6 is involved in this potassium–CK growth connection. Similar examination for photosynthetic response supports this connection further. All of the examined potassium mutants exhibited a greater sensitivity to CK when grown on standard media. *skor* and *ntr1.5* both experienced a larger reduction in Fv/Fm when CK was present in the low K + condition (Figure 2). The *crf6* mutants exhibited the opposite response; when CK was present in the low K + media, the plants had an increase in Fv/Fm levels. CRF6 represses the three K + transporter; it could be that without a functional CRF6 gene, these transporters are no longer repressed, making the low potassium media less stressful to the plants.

The altered *CRF6* lines themselves exhibited interesting patterns across the phenotypic analyses as compared to WT. Results from Fv/Fm analysis suggest that the *crf6* plants, when grown with sufficient potassium quantities, are more photosynthetically efficient than WT, in contrast, the addition of CK, which significantly reduces the *crf6* Fv/Fm levels below that of WT grown in the same condition (Figure 2). This exaggerated reduction in Fv/Fm readings with exogenous CK on standard media is a pattern mirrored in *crf6* growth data for roots (Figure 3) and shoots (Figure 4).

When *crf6* mutants were grown on reduced K + media, the Fv/Fm pattern was reversed from that measured on that with standard K+. On low K + media without CK treatment, the mutant Fv/Fm measurements were lower than those of WT, but with the addition of CK, the mutant Fv/Fm readings increased to nearly that of WT grown on reduced K + without CK (Figure 2). When the mutant plants were grown on low K + media, CK nearly rescued the mutant phenotype, restoring the measurements to levels similar to those observed in WT grown on the same media without the addition of CK.

Both trends, the larger changes in response to CK on standard media and the near phenotypic rescue on reduced potassium media in response to CK, were observed in the K + ionomics analysis of the shoots and roots of *crf6* plants (Figure 5). The connections among Fv/Fm, growth, and ionic content suggest a notable link among K+, *CRF6*, and overall plant function.

Although our testing conditions are designed to further investigate the microarray data, the conditions are not perfectly parallel; our transcriptomes were analyzed after a 6‐hr 2 µM CK treatment, whereas plants were grown on plates with 0.1 µM, and were left to grow on the media for 10 days. This is, in part, due to the difficulty of tracking CK‐based short‐term growth responses that would be similar to initial transcriptomic treatments. Long‐term exposure to exogenous CK, albeit at lower concentration, may have introduced additional unforeseen factors to these analyses. This could have obfuscated the effects of the K+‐poor environment, the transcriptional problems experienced in the mutant lines, and the effect of CK itself, as a long‐term continuing signal could cause enough stress to overwhelm other phenotypic responses. The *CRF6OE* line adds confounding factors itself because the gene is constitutively expressed throughout the entire plant, rather than only in the xylem, as CRF6 is natural, this creates additional complications in analyzing the data of the line. Furthermore, the K + mutants are a proxy for K transport. To that end, K + levels were directly examined in plants and specifically in roots versus shoots. Because the transporters are responsible not just for K + uptake, but also for making any K + present available to the rest of the plant, the K + levels of the root and aerial tissues were measured separately. The genes we examined are only part of a larger system. While *HAK5*, *NRT1.5,* and *SKOR* are very important to the transport and uptake of K+, they are not the only genes responsible for K + acquisition and movement. Similarly, CRF6 is not the whole of CK response and other CK‐regulated genes may be involved (ARR3 – as a CRF6‐CK DEG suggests that there is feedback to the TCS as an example). Despite these potential problems, the connections among CRF6, K+, and CK were still observed, which lends greater support to our hypothesis as we have been able to provide evidence for novel regulation of potassium transport mediated by CK through modulation of three important K transporters and CRF6, a key CK regulator of CK response.

## 4 MATERIAL AND METHODS

### 4.1 Plant materials and growth conditions

CRF6 mutant and altered transgenic lines (*crf6‐2*) and CRF6 overexpressor (19.3) were previously described in Zwack et al., 2016, while *hak5* (SALK_074868C)*, nrt1.5* (sail.72.FC.11), and *skor* (SALK.030047C) mutant lines were obtained from ABRC Stock Center. All lines are in the Col‐0 WT Arabidopsis background. All seeds that were grown on sterile media were surface sterilized in a solution of 70% ethanol for 10 min then a solution of 20% bleach and 0.5% tween solution, followed by 5 washes with sterile water. After the final wash, seeds were placed individually in rows onto media plates made with 0.8% agar and buffered to pH5.7 with MES contained full strength Murashige and Skoog (MS) medium, and 1% sucrose. Experimental growth media contained either MS or MS that was without potassium phosphate (“Murashige and Skoog (MS) Modified Medium w/o Potassium Phosphate” from Plantmedia, which contained monosodium phosphate rather than monopotassium phosphate) referred to as “low K+”, at the recommended concentration, both with and without 0.1 µM BA. After at least 24 hr at 4°C, the plated seeds were moved into a controlled environmental chamber and grown under standard conditions of 16 hr of 100 µE light at 22°C and 8 hr of darkness at 18°C.

### 4.2 Transcript expression analysis

Plants for this analysis were grown for 10 days under standard growth and media conditions. Seedlings were then lifted from the plates, transferred to 3 mM MES buffer pH 5.7, and allowed to gently shake for 1 hr. After which, 2 µM of CK (Benzyl Adenine or BA) was added or 0.2% DMSO vehicle control. Following 6 hr of treatment, plants were harvested and immediately frozen in liquid nitrogen. At least 10 individual seedlings were pooled from each line in each treatment group of two biological replicates.

RNA was isolated from the seedlings using the Qiagen RNAEasy Plant Mini kit according to the procedure specified by the manufacturer. Utilization of the Affymetrix Arabidopsis Gene 1.0 ST arrays and preprocessing of the data were performed by the Heflin Center for Genomic Science at the University of Alabama at Birmingham as a service. Normalization and differential expression analyses were performed using the FlexArray 1.6 software from McGill University and Genome Quebec. Fold change in expression was calculated by Cyber‐T analysis, and adjusted *p* values (*p*adj) were determined using the Benjamini Hochberg method of False Discovery Rate (FDR). Sequence data from this article can be found in the GenBank/EMBL data libraries under accession numbers GSE84770.

### 4.3 Real‐time qRT‐PCR verification of microarray DEGs

Plants for verification were sterilized and grown on 1xMS, 1% Sucrose, pH5.7 agar plates until day 10, then treated in a similar manner as for RNAseq analysis. qRT‐PCR verification was carried out using Sybr‐Green and sequence‐specific (listed below) primers. Reactions were carried out as previously described (Zwack et al., 2013). The following primers were used:

SKOR F, CCACGGATGTTCCTACCGAG, SKOR R, ACGCGGTTAATATGCGGGAA,

HAK5 F, CAGCTCAGAAGAGCCCATATG, HAK5 R, CCTAGATCAGCAAACATTGCC,

NRT1.5 F, GGGTTTTCTTTTTGGTTTGATG, NRT1.5 R, TATCTCGGTGTTCCAACAAGG, and the positive control, *tubulin 4*,

TUB F, ACCAATGAAAGTAGACGCCA, TUB R, AGAGGTTGACGAGCAAGATGA

### 4.4 Physiological Analyses (Shoot and Root Growth, F_v_/F_m_ assays)

Each of the three biological replicates conducted for the physiological analyses (except rosette measurements) contained at least 10 seedlings per line per treatment and was conducted when the plants were 14 days old, after 10 days of growing on the various media. Rosette diameter data were acquired from two biological reps with more than 10 seedlings per treatment per line.

Seeds of WT, *crf6‐2, CRF6OE, hak5, skor, and nrt1.5* lines were sterilized and sewn on standard media plates, then placed in 4°C conditions for 24 hr. Four days after the seeds were moved from 4°C conditions to the growth chamber under standard growing conditions, a minimum of 10 seedlings from each line were transferred to four types of experimental plates (Standard media, media + CK (0.1 µM BA), low K + media, and low potassium media + CK (0.1 µM BA)) and their root lengths were noted. The seedlings were then allowed to grow on these vertically aligned plates for 10 days, after which root growth was measured and the Fv/Fm assay was performed.

Root growth was measured from the noted initial root length to the end of the primary root and rosette diameter were both measured using ImageJ software (NIH).

Fv/Fm measurements were taken with a Handy FluorCam from Photon System Industries in a darkroom using Fluorcam 7 software and following the standard procedures to measure Fv/Fm as in Zwack et al., 2016. Plants to be examined were dark adapted for 30 m and Fv/Fm measurements performed as in Zwack et al., 2016. There were three biological replicates analyzed using Student's *t* Test.

### 4.5 Ionomics

For ionomic ICP‐OES measurements, the aerial tissue, “shoots” (including leaves, petioles, and the stem), and roots from 10‐day‐old plants as treated in the text were digested in 200 µL of concentrated nitric acid (Optima) in semi‐sealed acid‐washed microcentrifuge tubes heated to 98°C for 1 hr. Digests were diluted to 1 ml with ultra‐pure metal‐free water, then analyzed by Inductively Coupled Plasma with Optical Emission Spectroscopy (ICP‐OES, Perkin Elmer 7100 DV, Waltham, MA) with simultaneous measurement of C, Ca, Co, Cu, Fe, K, Mg, Mn, Mo, Na, P, S, and Zn. Metal concentrations were determined by comparing emission intensities to a standard curve created from certified metal standards (SPEX, Metuchen, NJ). Individual readings are the average of two intensity measurements that varied by less than 5%. Repeated analysis of individual samples showed less than 5% variability. Each replicate was normalized to the phosphorus measurements.

### 4.6 Statistical analyses

Statistical data are presented as the means of two to three biological replicates ± *SD*. one‐factor ANOVA, followed by post hoc, tests were used in combination with two‐tailed Student's *t* tests to determine significance among treatment groups, tissue types, and lines. ANOVA was used when comparing data of five or more groups and was followed by post hoc tests for specific comparisons. When comparing two sets of data within a dataset of 4 or fewer means, for example, comparisons made within a line between the two treatments without exogenous cytokinin, a two‐tailed Student's *t* test was used to determine the statistical significance of observed differences.

## CONFLICT OF INTEREST

The authors declare no conflict of interest.

## AUTHOR CONTRIBUTIONS

AMH and AMR wrote the study. AMH, PJZ, and AMR designed the experiments and analyzed the data. AMH and PJZ conducted the experimentation and collected the data. PAC provided instrumentation and instructed and assisted in all ICP‐OES ionomic analysis.

## Supporting information

Table S1

Table S2
